# Mechanisms of Iron Uptake from Ferric Phosphate Nanoparticles in Human Intestinal Caco-2 Cells

**DOI:** 10.3390/nu9040359

**Published:** 2017-04-04

**Authors:** Antonio Perfecto, Christine Elgy, Eugenia Valsami-Jones, Paul Sharp, Florentine Hilty, Susan Fairweather-Tait

**Affiliations:** 1 Norwich Medical School, University of East Anglia, Norwich, Norfolk NR4 7UQ, UK; a.perfecto@uea.ac.uk; 2 School of Geography, Earth and Environmental Sciences, University of Birmingham, Edgbaston, Birmingham B15 2TT, UK; c.n.elgy@bham.ac.uk (C.E.); e.valsamijones@bham.ac.uk (E.V.-J.); 3 Diabetes and Nutritional Sciences Division, King’s College London, London SE1 9NH, UK; paul.a.sharp@kcl.ac.uk; 4 Institute of Food, Nutrition, and Health, ETH, Schmelzbergstrasse 9, 8092 Zürich, Switzerland; florentine.hilty@gmail.com

**Keywords:** nano iron, NP-FePO4, bioavailability, Caco-2 cells, simulated gastrointestinal digestion, DMT1, endocytosis

## Abstract

Food fortification programs to reduce iron deficiency anemia require bioavailable forms of iron that do not cause adverse organoleptic effects. Rodent studies show that nano-sized ferric phosphate (NP-FePO_4_) is as bioavailable as ferrous sulfate, but there is controversy over the mechanism of absorption. We undertook in vitro studies to examine this using a Caco-2 cell model and simulated gastrointestinal (GI) digestion. Supernatant iron concentrations increased inversely with pH, and iron uptake into Caco-2 cells was 2–3 fold higher when NP-FePO_4_ was digested at pH 1 compared to pH 2. The size and distribution of NP-FePO_4_ particles during GI digestion was examined using transmission electron microscopy. The d50 of the particle distribution was 413 nm. Using disc centrifugal sedimentation, a high degree of agglomeration in NP-FePO_4_ following simulated GI digestion was observed, with only 20% of the particles ≤1000 nm. In Caco-2 cells, divalent metal transporter-1 (DMT1) and endocytosis inhibitors demonstrated that NP-FePO_4_ was mainly absorbed via DMT1. Small particles may be absorbed by clathrin-mediated endocytosis and micropinocytosis. These findings should be considered when assessing the potential of iron nanoparticles for food fortification.

## 1. Introduction

Iron deficiency is the most prevalent nutritional deficiency in the world, affecting 1–2 billion people worldwide [1]. Food fortification is an effective strategy to reduce the burden of iron deficiency in populations [2,3]. Iron salts, iron chelates, and elemental powders are all iron sources approved for food fortification [3], but each form has inherent disadvantages. Soluble iron compounds, such as ferrous sulfate (FeSO_4_) and ferrous gluconate, have high iron bioavailability but may cause adverse organoleptic changes when added to foods. Insoluble iron compounds, such as electrolytic iron and ferric phosphate (FePO_4_), are stable in foods but have low iron bioavailability [4,5,6]. FePO_4_ is an iron compound that causes no adverse organoleptic changes in food matrices, but is poorly absorbed (25%) relative to FeSO_4_ [4], therefore its nutritional value is limited.

The bioavailability of elemental iron powders has been shown to be inversely related to particle size. Harrison et al. [7] found that decreasing the particle size of FePO_4_ from 12–15 µm to 1 µm increased iron solubility and improved relative biological value (RBV) 5-fold. Decreasing particle size to the nanoscale could be a strategy to improve iron bioavailability. Recently, FePO_4_ synthesized to the nanoscale (NP-FePO_4_) by flame spray pyrolysis (FSP) was reported to have similar iron bioavailability to FeSO_4_ in rat models [8]. This has been attibuted to its increased solubility as a result of higher surface area relative to its larger precursors. However, further studies are required to confirm that the findings in rats can be extrapolated to humans [9] given that rodents endogenously synthesize ascorbic acid [10] and are less affected by dietary inhibitors of iron absorption than humans [11]. In the present study, we examined the effects of in vitro simulated gastrointestinal digestion on the size distribution of NP-FePO_4_, supernatant iron, uptake into Caco-2 cells, and mechanisms of absorption.

## 2. Materials and Methods

Samples of NP-FePO_4_, previously characterized for crystalline structure, phase distribution, chemical composition, and specific surface areas (SSA, 100 and 200 m^2^/g) [8,12], were kindly donated by ETH Zurich.

### 2.1. NP-FePO_4_ Treatments: Sonication

NP-FePO_4_ was diluted to a concentration of 1 mg/mL either in water or minimum essential media (MEM). The suspensions were probe sonicated for 15 min (MSE Soniprep 150 Plus, London, UK) on maximum setting (Amplitude: 16.1 A, 150 W).

### 2.2. NP-FePO_4_ Treatments: In Vitro Simulated Gastrointestinal (GI) Digestion

NP-FePO_4_ was exposed to in vitro simulated gastrointestinal digestion [13,14,15]. Briefly, 10 mL 40 mM NaCl and 5 mM KCl pH 2 solution containing 0.4% pepsin was added to NP-FePO_4_ in a 50 mL polypropylene tube. The mixture was placed on a rotating table (100 rpm) and incubated for 1 h at 37 °C. After 1 h, the pH of the digests was readjusted to 5.5–6.0 and a pancreatin-bile solution (0.25%) added. The pH was further adjusted to 6.9–7.0 with 1 M NaHCO_3_ and incubated for a further 30 min at 37 °C.

### 2.3. Transmission Electron Microscopy (TEM)

TEM micrographs were generated for visualization of NP-FePO_4_ (200) during in vitro digestion. During each digestion phase, an aliquot of digested NP-FePO_4_ (200) was drop spotted onto carbon-coated copper TEM mesh grids and dried for 1 h. TEM mesh grids were washed 5 times with milliQ H_2_O and subsequently air-dried for 24 h. Particles were visualized using a Jeol 1200EX electron microscope (JEOL, Tokyo, Japan) operated at 80 kV.

### 2.4. Centrifugal Disc Sedimentation

A CPS disc centrifuge model 2400 (CPS Instruments, Oosterhout, UK) was used to measure the total particle distribution given the heterogeneity, polydispersity, and aggregated/agglomerated particle patterns during in vitro digestion. The instrument was run at 5000 rpm (20 °C) for 30 min. A sucrose gradient was established using two sucrose solutions (24% and 8% *w*/*v*). After setting of the gradient, the instrument was calibrated to polystyrene standards (0.239 μm) followed by sample (100 µL).

### 2.5. Dynamic Light Scattering (DLS)

Hydrodynamic particle size of NP-FePO_4_ in cell culture media was measured using dynamic light scattering (DLS) with the Zetasizer Nano-ZS (Malvern Instruments, Malvern, UK). Particles (1 mg/mL in MEM) were sonicated and analysed immediately. The refractive index of the material was considered as 1.68. Intensity-based particle sizes were converted and are reported as volume based [16].

### 2.6. Caco-2 Cell Culture

Caco-2 cells (HTB-37^®^ VA) from the American Type Culture Collection were grown in Dulbecco’s Modified Eagle Medium (DMEM) supplemented with 10% fetal bovine serum, 1% non-essential amino acid solution, 1% penicillin/streptomycin, and 1% l-glutamine. Cells were grown in 75 cm^2^ cell culture flasks (Greiner, UK) and incubated in 5% carbon dioxide/95% air atmosphere at constant humidity. The media was replaced every 2–3 days. For iron uptake experiments, cells were seeded onto collagen-coated 6- or 12-well plates and grown for 12 days. After 12 days, cells were placed in MEM containing 19.4 mM D-glucose, 26.2 mM NaHCO_3_, 10 mM PIPES (piperazine-*N*,*N*-bis-(2-ethanesulfonic acid)), 1% antibiotic-antimycotic solution, 4 mg/L hydrocortisone, 5 mg/L insulin, 0.02 µM Na_2_SeO_3_, 0.05 µM triiodothyronine and 0.2 mg epidermal growth factor for 24 h prior to iron treatments. Experiments were conducted using cell passages 25–40.

### 2.7. Supernatant Iron during In Vitro Gastric Digestion

Supernatant iron from NP-FePO_4_ (soluble and <100 nm fractions) was quantified after iron dissolution at low pH [12,17]. Supernatant iron was measured with respect to time and pH at the end of the gastric phase. At each time point, 1 mL aliquots were removed from digestion and centrifuged immediately for 5 min at 11,600× *g*. The supernatant was removed and analyzed for iron content against a non-centrifuged control (total iron). The iron content of samples was determined using the iron chelator, 3-(2-pyridyl)-5,6-bis(5-sulfo-2-furyl)-1,2,4-triazine, disodium salt trihydrate (Ferene-S, Sigma, Gillingham, UK) with ammonium iron (II) sulfate as the iron standard curve. Briefly, 100 μL sample was diluted in 100 μL 1% HCl and incubated at 80 °C for 10 min. Samples were cooled on ice, followed by sequential addition of 500 μL 7.5% ammonium acetate, 100 μL 2.5% sodium dodecylsulphate, 100 μL 4% *w/v* ascorbic acid, and 100 μL 1.5% ferene. Samples were read at 593 nm.

### 2.8. Measurement of Iron Uptake into Caco-2 Cells

Iron uptake into Caco-2 monolayers was determined using cell ferritin formation (ng cell ferritin/mg cell protein). In each cell culture experiment, ferric ammonium citrate (FAC) was included as a control. FAC is a well-absorbed form of iron in Caco-2 cells and used as the reference for DMT1 uptake [18,19,20]. Reference blanks (cells not treated with iron) were included in each experiment to ensure low baseline levels of cell ferritin. After iron treatment, cells were washed twice with PBS and lysed with 200 μL CelLytic M protein lysis buffer (Sigma). Lysed cells were centrifuged (14,000× *g*, 15 min), supernatants collected, and analyzed for cell ferritin using the Spectroferritin ELISA kit (ATI Atlas, Chichester, UK). Cell ferritin was normalized to total protein concentrations using the BCA protein assay kit (ThermoFisher Scientific, Loughborough, UK).

### 2.9. Chemical Inhibitors Targeting Iron Uptake

All chemicals, unless otherwise stated, were from Sigma and used at the following concentrations. For in vitro simulated GI digestion experiments, ascorbic acid (AA) (1:20 Fe:AA molar ratio, 600 µM) was added at the beginning of the gastric phase as an enhancer of non-haem iron bioavailability. 2.5 mM CaCl_2_ (after dilution with digests) was added directly in MEM and used as an inhibitor of non-heme iron bioavailability. In both conditions, digests were incubated with cells for 24 h. For sonicated NP-FePO_4_ (200), clathrin-mediated endocytosis was inhibited using chlorpromazine hydrochloride (100 μM) and sucrose (0.5 M). Clathrin-independent endocytosis targeting caveolae-mediated endocytosis and macropinocytosis were inhibited with filipin (5 mg/L) and dimethyl amiloride (DMA, 200 μM), respectively [21,22,23,24,25,26]. Caco-2 cells were co-incubated with iron treatment and endocytosis inhibitors for 1 h, removed, and incubated for a further 23 h.

### 2.10. siRNA Knockdown of DMT1 in Intestinal Epithelial Cells

Caco-2 cells, seeded into 12-well collagen-coated plates (200,000 cells/well), were grown for 10 days. Transient knockdown of DMT1 was initiated according to the method of Latunde-Dada et al. 2014 [27]. Cell monolayers were transfected with Silencer^®^ Select siRNA targeting *SLC11A2* (the gene encoding DMT1) or Negative control no. 1 (200 nM, Life Technologies) using Lipofectamine 3000 in Opti-MEM (Gibco) for 48 h. After 48 h, siRNA complexes were replaced with FAC or NP-FePO_4_ (200) for 2 h. Iron treatments were removed, MEM added, and cells were incubated for a further 22 h. Wells in parallel with the same treatments were used to analyze for cell ferritin/protein and RNA extraction prior to RT-PCR. For Hutu-80 cells, 12-well plates (100,000 cells/well) were grown until 50%–70% confluent. Cell monolayers were transfected with Silencer^®^ Select siRNA targeting *SLC11A2* or Negative control no. 1 (10 nM) in Opti-MEM for 48 hours. Iron treatments and incubations paralleled the siRNA knockdown experiments undertaken in Caco-2 cells. Cell ferritin formation was normalised to FAC for siRNA experiments.

### 2.11. RT-PCR

The RNeasy Mini Kit (Qiagen, Hilden, Germany) was used for RNA extraction according to manufacturer’s instructions. RNA quality was determined using UV-Vis Nanodrop 2000 spectrophotometer (ThermoFisher Scientific, Loughborough, UK). Complementary DNA (cDNA) was synthesized using the qPCRBIO cDNA Synthesis Kit (PCR Biosystems, London, UK). 0.1 mg RNA was reverse transcribed to cDNA. Predesigned primers (KiCqStart SYBR Green Primers, Sigma, Gillingham, UK): *SLC11A2*, Forward: GAG TAT GTT ACA GTG AAA CCC; Reverse: GAC TTG ACT AAG GCA GAA TG; 18S, Forward: ATC GGG GAT TGC AAT TAT TC; Reverse: CTC ACT AAA CCA TCC AAT CG. Real time PCR proceeded with 4 μL cDNA and 2× SYBR Green Mix Lo-ROX (PCR Biosystems) and run using the Roche Lightcycler 480 (Roche, Burgess Hill, UK). Relative expression of *SLC11A2* (DMT1) was normalised to the housekeeping gene 18S, and assessed using the ∆∆Ct method [28].

### 2.12. Statistical Analysis

Statistical analysis was performed using GraphPad Prism v.6.0 (San Diego, CA, USA). Particle size was calculated using Feret’s diameter and particle size distributions expressed using the median particle size (d50) with d10 representing 10% and d90 representing 90% of the particle sizes. One-way repeated measures ANOVA with Tukey’s multiple comparisons test was used to compare differences in iron uptake or one-way repeated measures ANOVA with Dunnett’s test were used to compare differences between NP-FePO_4_ (200) and NP-FePO_4_ (200) treated with chemical inhibitors. Cell culture experiments were repeated 2–3 times, with *n =* 3 per experiment. Differences were considered significant at *p <* 0.05.

## 3. Results

### 3.1. Particle Size

#### 3.1.1. Characterization of Sonicated NP-FePO_4_

**Figure 1:**
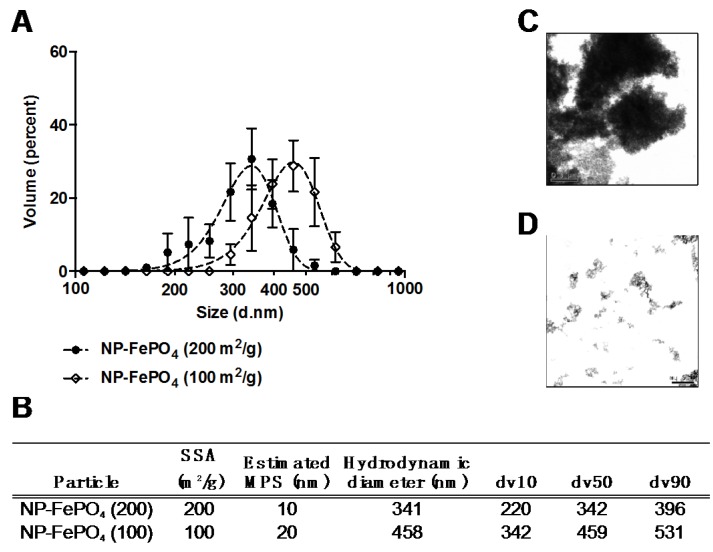
Size determination of sonicated nano-sized ferric phosphate (NP-FePO_4)_. 1 mg/mL NP-FePO_4_ dispersions in minimum essential media (MEM) were measured using dynamic light scattering, *n* = 3 (**A**,**B**). 1 mg/mL NP-FePO_4_ (200) directly diluted in H_2_O (unsonicated) (**C**) or dispersed by sonication and visualized using transmission electron microscopy (TEM) (**D**). SSA, specific surface areas; MPS, mean particle size.

**Figure 2:**
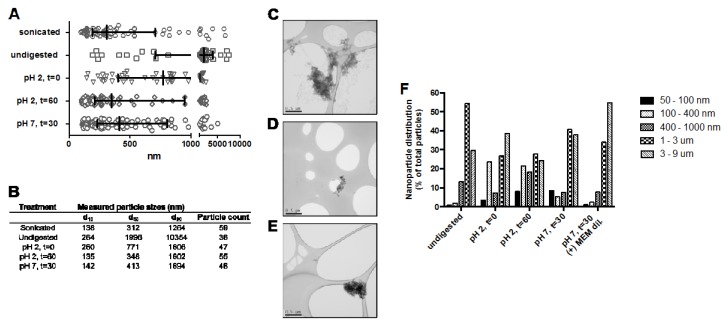
Size determination of NP-FePO_4_ (200) during different stages of in vitro digestion. 1 mg/mL stock solutions in water were diluted to 30 µg/mL in digestion solutions, and visualised using TEM. Particle sizes were calculated using ImageJ software analysis (**A**) and particle size distributions reflected in the table (**B**). Representative TEM micrographs used in the analysis of size and size distributions at pH 2, *t* = 0 min (**C**), pH 2, *t* = 60 min (**D**), and pH 7, *t* = 30 min (**E**). Histogram of particle distribution based upon total particle population during stages of in vitro digestion (**F**).

Sonicated NP-FePO_4_ (200) and NP-FePO_4_ (100) particle sizes were characterized in MEM using DLS. Sonicated NP-FePO_4_ (200) hydrodynamic diameter averaged 341 nm (d10, d90: 190, 459) and NP-FePO_4_ (100), 458 nm (d10, d90: 342, 532) (Figure 1A,B). Visual morphology of NP-FePO_4_ (200) comparing diluted (non-sonicated) or dispersed (sonicated) particles was conducted using TEM with water as the diluent. Large, agglomerated, electron dense particles formed without sonication in the micron range (Figure 1C) with d50 = 1990 nm (Figure 2B). Sonication of NP-FePO_4_ (200) resulted in particle dispersal of similar size to the acquired DLS data (Figure 1D); d50 = 312 nm.

#### 3.1.2. In Vitro Simulated GI Digested NP-FePO_4_ (200)

Particle size of NP-FePO_4_ (200) during in vitro digestion was visualized using TEM and particle size analysis with ImageJ software. Non-digested particles agglomerated in water as previously shown, resulting in d50 = 1996 nm. The particle size shifted to d50 = 771 nm after exposure to initial gastric digestion (pH 2). 60 min exposure in pH 2 caused further reduction of particle size to d50 = 348 nm. The particle size slightly agglomerated to d50 = 413 nm after the in vitro digest was neutralized (pH 7, *t =* 30) with 0.1 M NaHCO_3_ (Figure 2A,B). Total particle population size distibutions during in vitro digestion were conducted using CPS (Figure 2F). After 60 min digestion at pH 2, 50% of the particle sizes were ≤1000 nm, and 30% of the particles were ≤400 nm. At pH 7, rapid agglomeration of the particles led to an 80% increase in micron-sized fractions, and the proportions of the 100–400 nm and 400–1000 nm fractions were reduced to <10%. Overall, about 15% of the particles were ≤400 nm after in vitro digestion.

### 3.2. Cell Culture Experiments

#### 3.2.1. Effect of Gastric pH and Time on Supernatant Iron and Uptake into Caco-2 Cells

**Figure 3:**
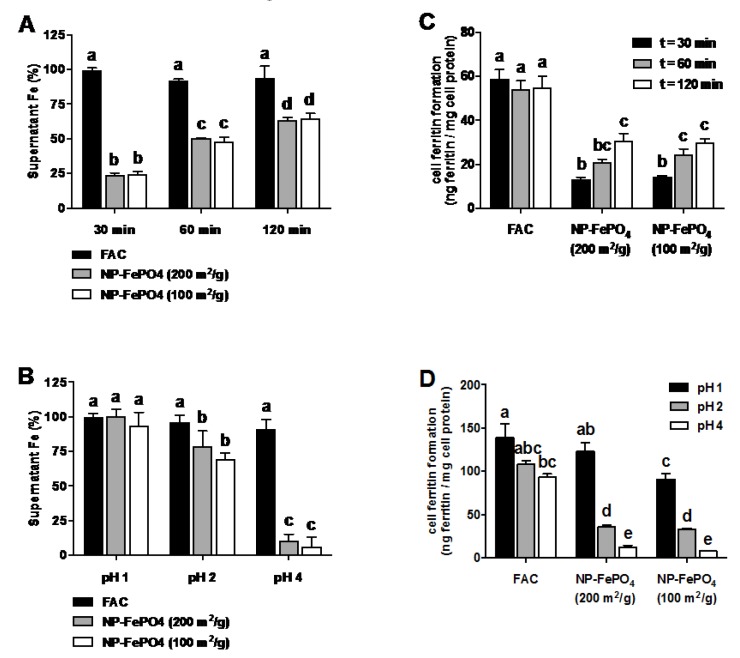
Effect of gastric digestion on supernatant iron and iron uptake in Caco-2 cells. Ferric ammonium citrate (FAC) or NP-FePO_4_ (30 µM Fe) was digested at pH 2 for varying times (**A**) or digested at varying pH for 1 hour (**B**) and supernatant iron determined at the end of gastric digestion. Caco-2 cells were exposed to digested FAC or NP-FePO_4_ (30 µM Fe) for 24 h and measured for cell ferritin formation (**C**,**D**). Data values are expressed as the means of two independent experiments (*n* = 3 per experiment, ± standard error of the mean (SEM)). One-way repeated measures analysis of variance (ANOVA) with Tukey’s multiple comparisons test was used to compare differences in iron uptake between iron treatment (FAC or NP-FePO_4_) with time or pH. Different letters indicate statistically significant differences (*p* < 0.05).

The effects of time of exposure and pH on supernatant iron concentration and uptake were compared using Caco-2 cells. At pH 2, NP-FePO_4_ (100 and 200) was digested for 30, 60, and 120 min and aliquots were taken to determine supernatant iron. The remaining digestion solution was neutralized at pH 7 and incubated for a further 30 min for cellular iron uptake. Supernatant iron increased with longer gastric digestion times, but no significant difference was observed between the two particle sizes. Compared to FAC, NP-FePO_4_ supernatant iron reached 70% after 2 h digestion (Figure 3A). Iron uptake was significantly increased when NP-FePO_4_ was digested for 120 min compared to 30 min for both particle sizes but only half as absorbed compared to FAC (Figure 3C). In contrast, the pH of the gastric digest had a large effect on supernatant iron and uptake of iron from NP-FePO_4_. Iron was completely in the supernatant fraction when NP-FePO_4_ was digested at pH 1, whereas 5%–10% of iron was in the supernatant at pH 4 for both particles (Figure 3B). NP-FePO_4_ was not taken up (compared to controls not containing iron) after digesting both particles at pH 4 for 1 h. Iron uptake from NP-FePO_4_ (200) was significantly increased relative to NP-FePO_4_ (100) when digested at pH 1 and similar to FAC (Figure 3D).

#### 3.2.2. Effect of AA and Calcium on Iron Uptake in Caco-2 Cells

**Figure 4:**
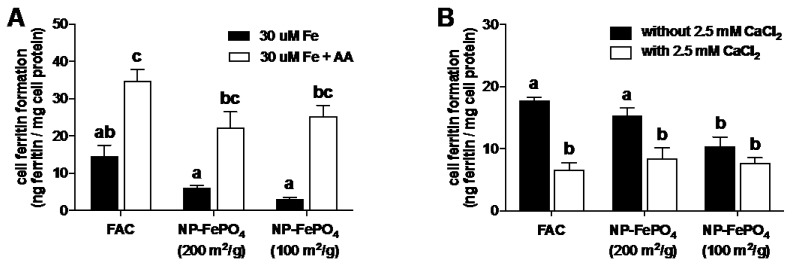
Iron uptake of in vitro simulated GI digested FAC or NP-FePO_4_ after incubation with ascorbic acid (1:20 AA molar ratio) introduced at the gastric phase or CaCl_2_ (2.5 mM, final volume in MEM after dilution) in Caco-2 cells after 24 h. Caco-2 cells were exposed to digested FAC or NP-FePO_4_ (30 µM Fe) and incubated with ascorbic acid (**A**) or CaCl_2_ in MEM (**B**) for 24 h and measured for cell ferritin formation. Data values are expressed as the means of two independent experiments (*n* = 3 per experiment, ± SEM). One-way repeated measures ANOVA with Tukey’s multiple comparisons test was used to compare differences in iron uptake between iron treatment (FAC or NP-FePO_4_) with enhancer (AA) or inhibitor (CaCl_2_). Different letters indicate statistically significant differences (*p* < 0.05).

NP-FePO_4_ was digested using standard in vitro gastrointestinal conditions. Particles were digested at pH 2 for 1 h and pH 7 for 30 min. Ascorbic acid added to the digest (1:20 AA molar ratio) during the gastric phase increased iron uptake of FAC 2-fold and both forms of NP-FePO_4_ 3–4 fold. The increase in iron uptake of NP-FePO_4_ with added AA was similar to FAC and FAC with AA (Figure 4A), correlating with increased supernatant iron of NP-FePO_4_ with AA at neutral pH (Supplementary Figure S1). NP-FePO_4_ was digested and added to MEM containing a final concentration of 2.5 mM CaCl_2_ after dilution (Figure 4B). Calcium (as CaCl_2_) inhibited iron uptake from FAC and NP-FePO_4_ (200) by 50%. Iron uptake of NP-FePO_4_ (100) was marginally decreased with CaCl_2_ but the effect was not statistically significant, which might be due to the low basal levels of iron uptake observed (Figure 4B).

**Figure 5:**
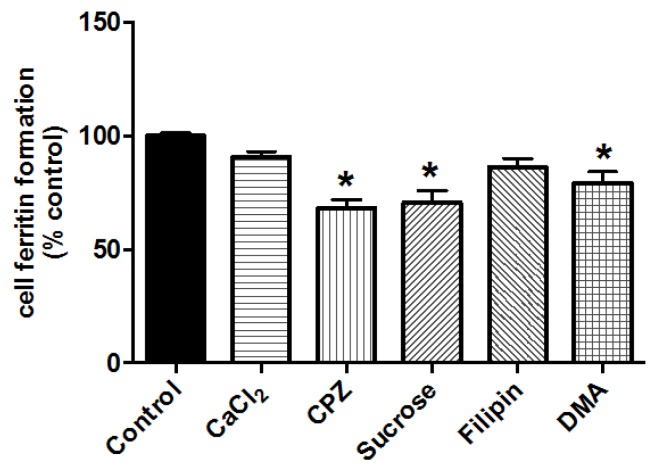
Iron uptake of sonicated NP-FePO_4_ (200) co-incubated with chemical inhibitors targeting endocytosis uptake pathways in Caco-2 cells as measured by cell ferritin formation. NP-FePO_4_ (200) (100 µM Fe) was incubated directly in MEM with inhibitors and placed onto Caco-2 cells for 1 h. Cells were washed 3× with PBS and replaced with MEM for a further 23 h. Data values are expressed as the means of two independent experiments (*n* = 3 per experiment, ± SEM). One-way repeated measures ANOVA with Dunnett’s multiple comparison test was used to distinguish differences in iron uptake between control (NP-FePO_4_ (200)) and NP-FePO_4_ (200) co-incubated with chemical inhibitors. Asterisks indicate statistically significant differences from control (*p* < 0.05).

Using sonicated NP-FePO_4_ (200), CaCl_2_ did not inhibit iron uptake. Iron uptake was significantly reduced when sonicated particles were incubated in the presence of chlorpromazine, sucrose, and DMA (Figure 5).

#### 3.2.3. Effect of siRNA-Mediated Knockdown of DMT1 in Caco-2 and Hutu-80 Cells

**Figure 6:**
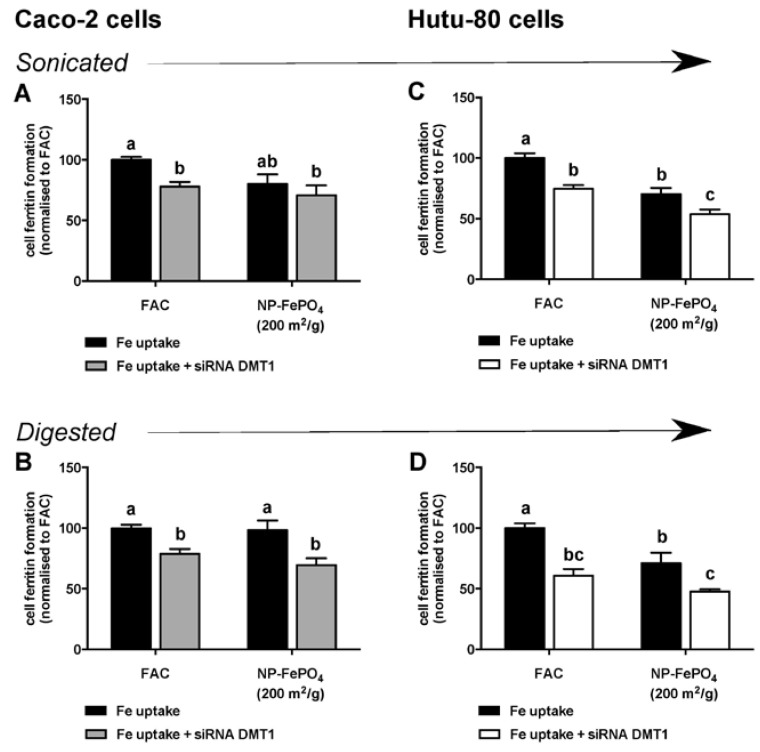
Iron uptake of sonicated and in vitro digested NP-FePO_4_ (200) or FAC in non-targeting siRNA (negative control 1) or *SLC11A2* siRNA treated Caco-2 cells (**A**,**B**) or Hutu-80 cells (**C**,**D**) after 2 h exposure followed by incubation in MEM for a further 22 h. Cell ferritin formation was normalized relative to FAC treatments. Data values are expressed as the means of three independent experiments (*n* = 3 per experiment, ± SEM). One-way repeated measures ANOVA with Tukey’s multiple comparisons test was used to compare differences in iron uptake between iron treatment (FAC or NP-FePO_4_ [200]) in non-targeting siRNA or *SLC11A2* siRNA treated cells. Different letters indicate statistically significant differences (*p* < 0.05).

Under our experimental conditions, DMT1 expression was reduced by 20% in Caco-2 cells following exposure to *SLC11A2* siRNA (Figure 6A). The reduction in DMT1 expression was sufficient to significantly reduce iron uptake from FAC by 20%. Iron uptake from sonicated NP-FePO_4_ (200) was not different in siRNA DMT1 treated cells (Figure 6B) relative to control cells. However, uptake from digested NP-FePO_4_ (200) was decreased in DMT1-knockdown cells (Figure 6C).

The Hutu-80 cell line was used to validate DMT1 knockdown of Caco-2 cells, since its transfection efficiency is improved in comparison to Caco-2 cells. DMT1 expression was reduced by 50% in Hutu-80 cells. In both digested and sonicated NP-FePO_4_ (200), DMT1 knockdown resulted in significant inhibition of iron uptake by 40%–50% (Figure 6C,D). In both cell lines, DMT1 expression from negative control siRNA was not different than from control cells without siRNA.

## 4. Discussion

Food iron fortification is technically challenging as the fortificant cannot react with food matrices. The elemental powders H-reduced, carbonyl, electrolytic iron, FePO_4_, and ferric orthophosphate do not react with food matrices and are widely used. However, low iron bioavailability of elemental powders relative to water-soluble compounds limit their nutritional value. Particle size reduction is an effective strategy for improving iron bioavailability from elemental powders [29]. Particle size reduction is inversely related to iron solubility at low pH, which has been shown to be the best in vitro predictor of iron bioavailability in humans [17,30]. The advent of nanotechnology has facilitated the synthesis of “micro” and “nano” sized particles with defined size and morphology. Recent studies have demonstrated good iron bioavailability of micronized ferric pyrophosphate (SunActive© Fe, Yokkaichi Japan), with estimated mean particle size of 0.3 µm, in humans [31,32,33,34]. The bioavailability of NP-FePO_4_ has been shown to be effective as ferrous sulfate in rats using the hemoglobin repletion assay [8,12]. The present study aimed to elucidate the mechanisms involved in iron uptake from NP-FePO_4_ using cellular models.

We investigated two separate mechanisms of iron uptake into Caco-2 cells; iron uptake via DMT1, and direct endocytosis of NP-FePO_4_. We first characterised NP-FePO_4_ in solution. Nanoparticle toxicity is a concern since small particle size is a factor that can lead to enhanced rates of translocation into body tissues. With increasing interest in using iron nanoparticles as food fortificants, it is possible that non-homeostatically regulated iron uptake may occur, resulting in oxidative stress. An assessment of the toxicity of specific particles includes consideration of particle size, dispersity, agglomeration state, surface characteristics, charge, and cell type, as each parameter is an important factor determining nanoparticle uptake [35,36,37,38].

In addressing exposure of Caco-2 cells to NP-FePO_4_, we modelled the size distribution of particles during a physiologically relevant exposure using a validated in vitro method [13,39]. This method estimates iron bioavailability from an in vitro simulated GI digestion/Caco-2 cell model and has shown to correlate well with data from human trials [40,41]. Using disc centrifugal sedimentation (DCS), the majority of particles agglomerated when NP-FePO_4_ (200) was exposed to simulated in vitro digestion. Agglomerated particle fractions suggest limited toxicity of NP-FePO_4_ (200) to the intestinal epithelium with respect to its smaller (dried) precursors [11,21,27]. Agglomeration of particles was not observed using TEM, which is likely due to technical differences in microscopy and laser diffraction [28].

Next we examined the effects of gastric digestion on iron uptake of NP-FePO_4_ in Caco-2 cells. We use the term supernatant iron throughout, rather than soluble iron, as it accurately describes the nature of the aqueous phase containing a combination of soluble iron and nanoparticles <100 nm after centrifugation when 3 kDa molecular weight cut off (MWCO) spin filters are omitted [16,26]. Cell ferritin formation served as the surrogate marker of iron absorption [8,33]. Gastric digestion at pH 1 for 1 h significantly increased supernatant iron and iron uptake compared to pH 2 and pH 4. Gastric digestion at pH 2 for 30 min, 60 min, and 120 min led to increases in supernatant iron and uptake with time, but the effect of time was much less pronounced than pH. Coupling gastric digestion at pH 1 to Caco-2 cells resulted in more bioavailable iron from NP-FePO_4_ (200) compared to NP-FePO_4_ (100). Iron uptake of NP-FePO_4_ (200) was similar to FAC. Rohner et al. [6] and Hilty et al. [7] observed similar iron uptake of NP-FePO_4_ (200) compared to FeSO_4_ in rats and suggested that its high iron uptake is a result of increased iron particle dissolution compared to commonly used FePO_4_.

While iron bioavailability of foods and the effects of dietary factors using the in vitro digestion/Caco-2 cell model has been shown to correlate well with human absorption data [40,41], the usefulness of the model for elemental iron powders as a predictor for human bioavailability has been questioned [5,42]. Breads fortified with 8 micron sized H-reduced iron were more bioavailable than breads fortified with 45 micron sized H-reduced iron in Caco-2 cells, and a linear relationship was found between solubility and iron bioavailability [43]. Similarly, 40–60 nm H-reduced iron particles were more bioavailable compared to its larger precursors [44]. Others have found that the Caco-2 cell model cannot consistently predict the bioavailability of iron fortificants within food matrices that have been observed in humans [45,46]. Forbes et al. compared two iron fortificants, electrolytic iron and ferric orthophosphate, with FeSO_4_ using in vitro solubility at pH 1.2 and absorption studies in humans [4]. The compounds were ranked in the same order (ferrous sulfate > electrolytic iron > ferric orthophosphate), although the solubility test underestimated the relative biological value of ferric orthophosphate. They concluded that these results, together with those from earlier studies, suggested that solubility in dilute acid provides a screening method for assessing iron fortification compounds. However, pH 1.2 is non-physiological because it is lower than the pH found within the stomach. In our experiments, we observed that the supernatant iron concentration from NP-FePO_4_ at pH 1 was equal to that of FAC, and that there was a positive correlation with Caco-2 cell ferritin concentration; iron uptake of NP-FePO_4_ (200) was equal to that of FAC at pH 1. These results indicate that the dissolution of nano-particulate iron is a function of gastric pH.

We investigated the role of DMT1 transport in iron uptake from digested NP-FePO_4_ in Caco-2 cells. Two to 4-fold increases in ferritin formation were observed when AA was added to the gastric digest with NP-FePO_4_, similar to FAC. AA is a potent enhancer of non-heme iron absorption which occurs via reduction of Fe3+ to Fe2+ and solubility at intestinal pH [10,47]. Our data suggest that AA increases iron uptake of NP-FePO_4_, similar to FAC and FAC + AA in Caco-2 cells, by facilitating Fe^2+^ DMT1 mediated uptake. We further confirmed that iron uptake from NP-FePO_4_ occurred via DMT1 by incubating Caco-2 cells with 2.5 mM CaCl_2_. Calcium has been shown to inhibit non-heme iron uptake in Caco-2 cells by decreasing apical protein expression of DMT1 [20]. Iron uptake from in vitro digested FAC and NP-FePO_4_ was reduced when incubated with CaCl_2_, providing evidence that DMT1 is required for iron uptake. Using siRNA to target DMT1 in both Caco-2 and Hutu-80 cell lines demonstrated that iron uptake was decreased in DMT1 knockdown cells compared to control cells in both cell lines, thereby confirming the role of DMT1 in iron uptake from NP-FePO_4_.

DMT1 transport is most likely the predominant mechanism of iron uptake. We also speculated that an alternative route of iron uptake is also operational, given that ~15% of the particles were between 50 and 400 nm (Figure 2F). Gastric pH in rats is considerably higher than pH 1 [48,49] and our experiments showed that a large amount of iron (25%–50%) from NP-FePO_4_ remained insoluble at pH 2, with little iron in the supernatant fraction at pH 4. This suggests that a significant fraction of iron bound to NP-FePO_4_ could be directly transported into the epithelium by endocytosis, given particle translocation of 200 and 500 nm particles seen in Caco-2 cells co-cultured with M-cell like phenotype [36,50]. This was investigated using sonicated NP-FePO_4_. The particle size range of sonicated NP-FePO_4_ (200) was similar to the range expected for the non-agglomerated fraction of in vitro digested NP-FePO_4_ (200). Iron uptake from sonicated NP-FePO_4_ (200) was inhibited with chlorpromazine and sucrose, and to a lesser extent DMA (Figure 5). The chemical inhibitors used have been shown to be successful in inhibiting endocytic pathways in Caco-2 cells [24,25,26]. We did not observe an effect on iron uptake from sonicated NP-FePO_4_ with CaCl_2_, suggesting that uptake is independent of DMT1. This needs to be further clarified as excess calcium may cause particle agglomeration. Sonicated NP-FePO_4_ (200) had a wide particle distribution (150–500 nm), therefore we hypothesize that the smaller particles may be absorbed using clathrin-mediated endocytosis and the larger particles using macropinocytosis. In recent reports, TEM micrographs of nanoparticle uptake have been observed for iron compounds from 10–100 nm size in Caco-2 cells [26,51,52]. Moreover, in support of our findings, a number of previous studies have shown that nano-sized iron compounds are absorbed using endocytic pathways in Caco-2 cells [22,23,26]. This suggests that our data are not the result of increased transcytosis of NP-FePO_4_ (200) caused by toxic effects induced by iron treatment or chemical inhibitors (Supplementary Figure S2).

The long-term exposure and potential toxicity to NP-FePO_4_ require further investigation. Health concerns have been raised about the toxicological effects of daily consumption of nanoparticles found in various consumer products (see review by Nel et al. [35]). Although evidence from our study suggests that NP-FePO_4_ is non-toxic to Caco-2 cells after 24 h exposure, recent studies have shown that chronic exposure of polystyrene or TiO2 nanoparticles to Caco-2/HT-29 MTX co-cultures markedly remodel the intestinal epithelium and affect iron absorption [53,54].

The future use of NP-FePO_4_ as an iron fortificant is to some extent dependent on factors other than nutritional considerations. Food fortification for large-scale populations requires that the iron compound is cost-effective and scalable. Although inherent disadvantages exist in the use of FeSO_4_ as a fortificant, namely its rancidity/instability during storage and color induced changes to the matrix [5,7], its use meets the criteria described above compared to other iron compounds, such as NaFeEDTA and FePP [55]. Advances in nanotechnology necessitate decreasing the cost and increasing the scalability of production prior to its use as an iron fortificant.

## 5. Conclusions

Our experiments demonstrate that iron uptake from NP-FePO_4_ occurred predominately through increased iron in the supernatant fraction and entry via the DMT1 transporter in the Caco-2 cell model. Some NP-FePO_4_ (200) may be absorbed intact in Caco-2 cells independently from DMT1, but most likely this mechanism has a minor role in iron uptake. We reach this conclusion because our digestion experiments showed less than 15% of NP-FePO_4_ (200) remained as particles <400 nm, and in our sonicated NP-FePO_4_ (200) experiments, chlorpromazine and siRNA DMT1 treated cells inhibited 20% of iron uptake. Our results suggest that NP-FePO_4_ (200) endocytosis in Caco-2 cells is dependent on particle size, with clathrin-mediated endocytosis (CME) as the predominant mechanism of particle internalization. To our knowledge, this is the first paper suggesting a mechanism of particle transcytosis of iron compounds for food fortification. However, one of the assumptions made in our experiments is that sonicated particles are similar to the nanoparticle fractions after in vitro digestion, which illustrates the difficulties in translating the relatively novel field of nanotechnology to biological systems. Nevertheless, our experiments suggest that the iron uptake from NP-FePO_4_ (200) results from both DMT1 transport and particle transcytosis, which should be taken into consideration when assessing the potential of iron nanoparticles for food fortification.

## Supplementary Materials

The following are available online at www.mdpi.com/2072-6643/9/4/359/s1, Figure S1: Supernatant iron of digested NP-FePO_4_ after in vitro simulated GI digestion, Figure S2: Cell viability of Caco-2 cells measured 4 h or 24 h after incubation with chemical inhibitors or iron compounds.
