# Supplementary material for: Mechanisms of Iron Uptake from Ferric Phosphate Nanoparticles in Human Intestinal Caco-2 Cells
Source: Nutrients. 2017 Apr 4;9(4):359. doi: 10.3390/nu9040359 (PMC5409698; doi:10.3390/nu9040359)
Supplement: Supplementary file 1 [file nutrients-09-00359-s001.pdf]

Supplementary Material: Mechanisms of Iron Uptake from Ferric Phosphate Nanoparticles in Human Intestinal Caco-2 Cells

Antonio Perfecto, Christine Elgy, Eugenia Valsami-Jones, Paul Sharp, Florentine Hilty and Susan Fairweather-Tait

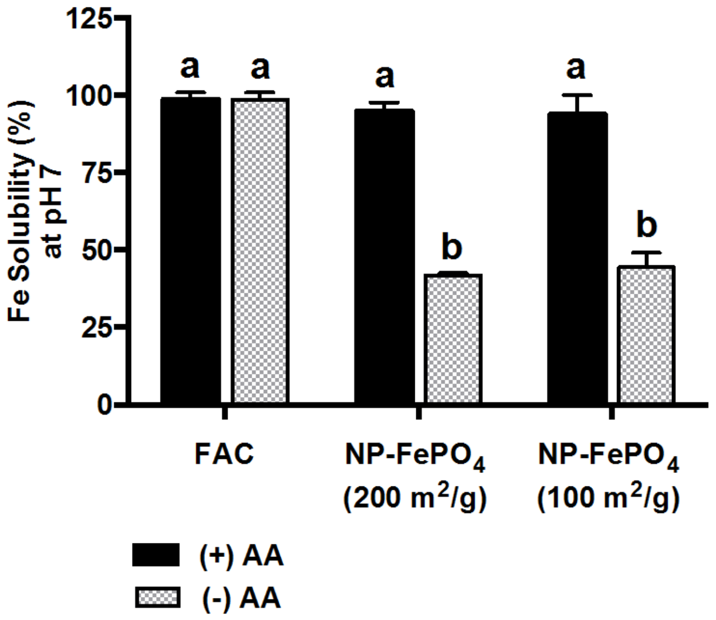

Figure S1. Iron solubility of digested NP-FePO4 after in vitro simulated GI digestion. NP-FePO4 was digested at pH 2 for one hour, neutralized to pH 7 with 1 M NaHCO3, incubated for another 30 min and iron solubility measured. Data values are expressed as the n = 3 ± SD. One-way ANOVA with Tukey's multiple comparison test was used to distinguish differences in iron bioavailability with AA. Different letters indicate statistically significant differences ($p < 0.05$).

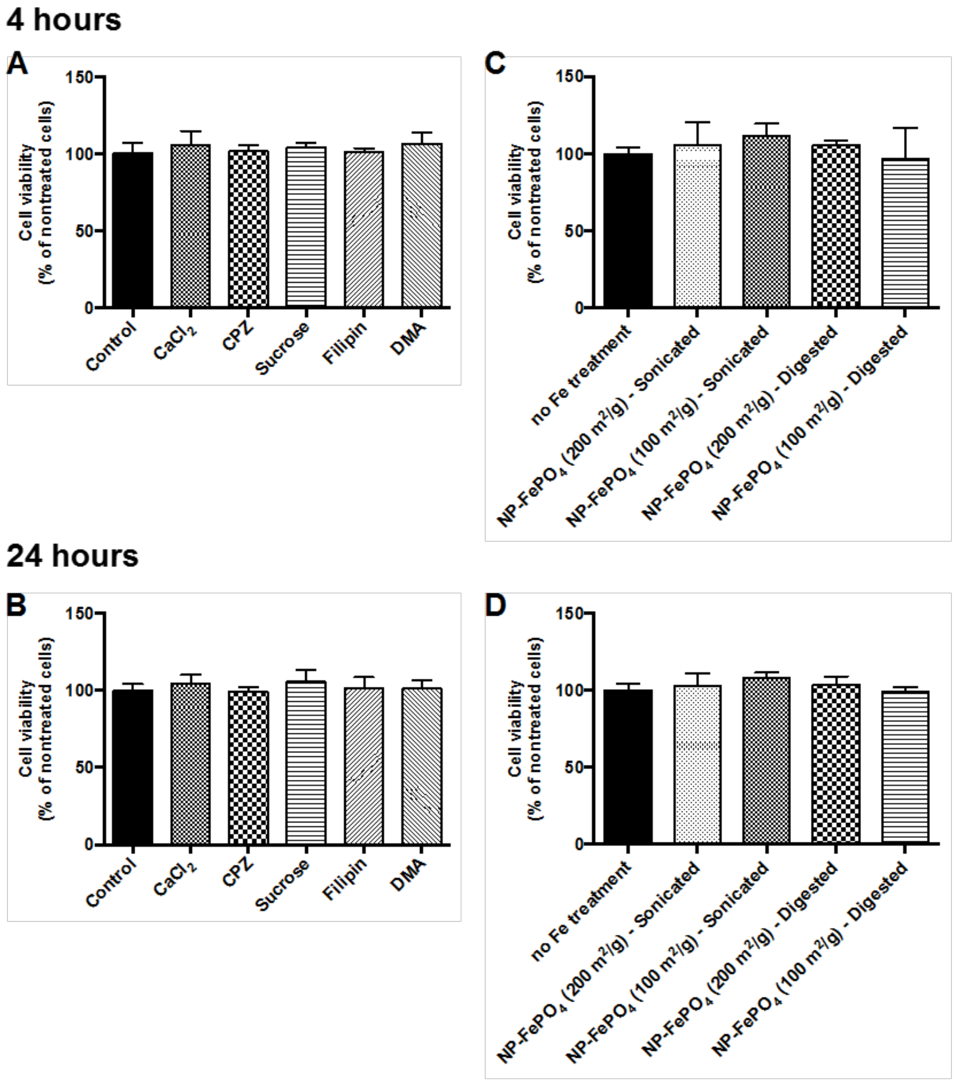

Figure S2: Cell viability of Caco-2 cells measured 4 hours or 24 hours after incubation with chemical inhibitors or iron compounds and assessed using the MTS assay (Promega, UK). Chemical inhibitors with concentrations used in the described experiments were incubated with Caco-2 cells for 1 hour (**A**, **C**). Iron compounds were incubated with Caco-2 cells for either 1 hour (100 μM Fe) or 24 hours (30 μM Fe) (**B**, **D**). 1% Triton X-100 was used as the positive control with 10-40% cell viability in each experiment. Data values are expressed as the n = 3 ± SD. One-way ANOVA with Dunnett's multiple comparison test was used to distinguish differences between untreated cells with chemical inhibitor or iron compound. Different letters indicate statistically significan differences ($p < 0.05$).

© 2017 by the authors. Licensee MDPI, Basel, Switzerland. This article is an open access article distributed under the terms and conditions of the Creative Commons by Attribution (CC-BY) license (http://creativecommons.org/licenses/by/4.0/).
